# Edible Fungi in Functional Aquafeeds: Prebiotic and Immunostimulatory Effects in Commercially Important Farmed Fish

**DOI:** 10.3390/ani16152419

**Published:** 2026-08-05

**Authors:** Marco Valdés, John Quiñones, Matias Cortes, Rodrigo Huaiquipán, Ailín Martínez, Erwin A. Paz, Néstor Sepúlveda, Rommy Díaz

**Affiliations:** 1 Programa de Doctorado en Ciencias Agroalimentarias y Medioambiente, Universidad de La Frontera, Temuco 4780000, Chile; m.valdes09@ufromail.cl (M.V.); m.cortes10@ufromail.cl (M.C.); r.huaiquipan01@ufromail.cl (R.H.); 2 Center for Innovation and Technology in Meat Quality (CTI-Carne), Universidad de La Frontera, Av. Francisco Salazar 01145, Temuco 4811230, Chile; a.martinez26@ufromail.cl (A.M.); nestor.sepulveda@ufrontera.cl (N.S.); 3 Faculty of Agricultural and Environmental Sciences, Universidad de La Frontera, Av. Francisco Salazar 01145, Temuco 4811230, Chile; 4 Doctoral Program in Science Major in Applied Cellular and Molecular Biology, Universidad de La Frontera, Av. Francisco Salazar 01145, Temuco 4811230, Chile; 5 UWA Institute of Agriculture, The University of Western Australia, Perth, WA 6009, Australia; erwin.pazmunoz@uwa.edu.au

**Keywords:** aquaculture, immunostimulants, edible fungi, β-glucans, gut microbiota

## Abstract

Aquaculture faces structural constraints linked to input sustainability, animal health, and a persistent reliance on fishmeal and fish oil, alongside the intensive use of antibiotics in high-density production systems. In this context, edible fungi have emerged as promising functional ingredients, primarily due to their content of structural polysaccharides, particularly β-glucans and chitin. This review synthesizes recent evidence on the dietary inclusion of fungal-derived compounds in aquafeeds for commercially relevant species. Overall, these bioactive components have been associated with beneficial effects on growth performance, feed efficiency, antioxidant status, and disease resistance in species such as *Oncorhynchus mykiss*, *Salmo salar*, and *Oreochromis* spp. However, the magnitude and consistency of these responses remain highly variable across studies. Such variability is primarily attributed to differences in the structural composition of the ingredients, their physicochemical form, processing methods, and their compatibility with host digestive physiology, all of which influence functional bioavailability and the resulting biological responses. Consequently, their biological effects are context-dependent and should be interpreted according to ingredient characteristics, host species, dietary inclusion level, and experimental conditions rather than assuming a universal dose–response relationship. Effective implementation will require advances in matrix standardization, detailed structural characterization of bioactive compounds, and validation under industry-relevant conditions.

## Simple Summary

Fish farming must increase production while reducing reliance on fishmeal, fish oil and antibiotics. Edible fungi, such as oyster, shiitake and reishi mushrooms, contain bioactive compounds that may support intestinal function, feed utilization, and innate immune responses in fish. This review examines how edible fungi and fungal-derived compounds, especially β-glucans and chitin, may contribute to gut microbiota modulation and immune stimulation in diets for commercially important fish. The available evidence indicates that their biological effects depend strongly on the fish species, ingredient form, dietary inclusion level, processing method and experimental conditions. Omnivorous fish such as tilapia generally tolerate broader inclusion levels, whereas carnivorous salmonids appear to respond more consistently to low dietary levels of extracts or processed fractions. Therefore, edible fungi should be regarded as complementary functional ingredients rather than simple protein replacements, requiring species-specific formulation strategies, standardized ingredient characterization, and validation under commercial farming conditions.

## 1. Introduction

Over the past decades, aquaculture has become one of the fastest-growing food production sectors worldwide, playing a pivotal role in global food security, nutrition, and sustainable food systems. According to the latest Food and Agriculture Organization (FAO) State of World Fisheries and Aquaculture [1,2] report, total global fisheries and aquaculture production reached a record 235 million tons in 2024, including 195 million tons of aquatic animals. Aquaculture production of aquatic animals exceeded 103 million tons for the first time, accounting for approximately 53% of total aquatic animal production and nearly 60% of aquatic animal foods destined for direct human consumption. Furthermore, aquatic animal foods currently provide at least 20% of the animal protein intake for approximately 3.1 billion people worldwide, highlighting the strategic importance of aquaculture in addressing the growing global demand for nutritious and sustainable foods [1,2].

This rapid expansion has been accompanied by structural challenges related to feed ingredient sustainability, environmental impact, and health management in intensive production systems [3,4,5]. The continued reliance on fishmeal and fish oil, together with the recurrent use of antibiotics in some intensive aquaculture production systems, exerts increasing pressure on marine ecosystems, production costs, and the emergence of antimicrobial resistance [6,7,8,9]. Traditionally, aquafeed formulation has been primarily driven by the need to satisfy the nutritional requirements of cultured species through adequate supplies of protein, lipids, and energy [10]. However, increasing attention has recently been directed toward functional nutrition, which seeks to improve host physiological performance beyond basic nutritional requirements by enhancing intestinal health, immune competence, stress resilience, and disease resistance [11,12,13].

In parallel with the expansion of aquaculture, the global edible mushroom industry has experienced sustained growth due to increasing consumer demand, advances in controlled cultivation technologies, and the implementation of circular bioeconomy strategies that promote the valorization of agricultural and agro-industrial by-products. Edible fungi such as *Pleurotus ostreatus, Lentinula edodes*, *Agaricus bisporus*, and *Ganoderma lucidum* are among the most widely cultivated mushroom species worldwide, generating substantial quantities of fruiting bodies, mycelial biomass, spent mushroom substrate, and processing residues that represent promising and sustainable raw materials for aquafeed production [14,15,16,17]. Among the candidate functional ingredients, edible fungi have attracted considerable interest because of their favorable nutritional profile, which includes high-quality protein containing essential amino acids, dietary fiber, vitamins, minerals, relatively low lipid concentrations, and a wide diversity of biologically active compounds [14,15,16,17,18]. These bioactive constituents include β-glucans, chitin, mannoproteins, heteropolysaccharides, phenolic compounds, ergothioneine, terpenoids, lectins, peptides, sterols, and antioxidant metabolites, many of which have demonstrated immunomodulatory, antioxidant, antimicrobial, anti-inflammatory, and gut health-promoting properties in both terrestrial and aquatic animals [16,17,18,19,20].

Among these compounds, fungal β-glucans are among the most extensively characterized functional molecules. Unlike cereal β-glucans, which predominantly contain β-(1→3) and β-(1→4) glycosidic linkages, macrofungal β-glucans are mainly composed of β-(1→3)-linked glucose backbones with β-(1→6)-linked side branches. This distinctive molecular architecture largely determines their recognition by host pattern-recognition receptors (PRRs), thereby influencing downstream innate immune signaling pathways and contributing to their biological activity in fish and other vertebrates [17,18,19,20,21].

From a functional perspective, fungal polysaccharides may exhibit prebiotic potential by acting as selectively utilized substrates for beneficial microorganisms of the intestinal microbiota, thereby modulating microbial composition and metabolic activity [22,23,24]. According to the consensus definition proposed by the International Scientific Association for Probiotics and Prebiotics (ISAPP), a prebiotic is “a substrate that is selectively utilized by host microorganisms conferring a health benefit.” Therefore, not all fungal polysaccharides should automatically be considered true prebiotics. In many cases, the terms “prebiotic potential” or “prebiotic-like effects” more accurately describe the available experimental evidence unless selective microbial utilization and associated host benefits have been conclusively demonstrated [25,26,27]. These microbiota-mediated interactions are associated with the production of metabolites such as short-chain fatty acids (SCFAs), improved intestinal barrier function, and modulation of local and systemic immune responses, supporting the concept of an integrated gut–microbiota–immune axis [27,28,29,30].

Despite the increasing number of studies investigating edible fungi in aquaculture, the available evidence remains highly heterogeneous with respect to growth performance, physiological responses, immune modulation, antioxidant capacity, intestinal health, and microbiota composition across different fish species, fungal matrices, inclusion levels, and experimental conditions [14,31]. This variability suggests that the observed responses are influenced not only by methodological differences among studies but also by intrinsic biological factors associated with fungal composition, processing methods, host digestive physiology, and production conditions [11,32]. In many investigations, edible fungi have been considered simply as alternative protein ingredients without adequately distinguishing between whole mushroom meals, purified extracts, isolated polysaccharides, mycelial biomass, fermented substrates, fungal by-products, or mycoprotein, despite their markedly different nutritional characteristics and biological properties [18,33,34,35,36]. Likewise, although numerous mechanisms underlying their biological activity have been proposed, the current evidence remains fragmented and lacks a comprehensive framework capable of integrating nutritional, microbiological, immunological, and physiological responses across commercially important aquaculture species [11,14,18,31]. Consequently, important knowledge gaps remain regarding species-specific dietary inclusion strategies, ingredient standardization, mechanisms of action, and validation under commercial farming conditions, limiting the effective translation of experimental findings into practical aquaculture applications [34,35].

Previous reviews have primarily focused on the nutritional replacement potential or the immunostimulatory properties of fungal products individually, whereas the present review integrates nutritional composition, structural characteristics of fungal-derived bioactive compounds, gut microbiota interactions, host physiological responses, and practical implications for commercial aquaculture. The objective of this review is to critically evaluate the current scientific evidence regarding edible fungi and fungal-derived ingredients as functional components of aquafeeds, with particular emphasis on their nutritional composition, bioactive compounds, prebiotic potential, immunomodulatory properties, antioxidant activity, and species-specific physiological responses. Furthermore, this review aims to identify current knowledge gaps, assess the strength of the available evidence, and provide a practical framework to support future research and facilitate the responsible incorporation of edible fungi into commercial aquaculture production systems.

## 2. Methodology

### 2.1. Literature Search Strategy

This review was conducted as a critical narrative review supported by a structured literature search designed to identify, evaluate, and synthesize the available evidence regarding the use of edible fungi and fungal-derived ingredients as functional components of aquafeeds for commercially important fish species. The literature search was performed in Scopus, Web of Science Core Collection, PubMed, and Google Scholar, covering publications from January 2019 to July 2026. Earlier studies were included when they provided essential mechanistic, methodological, or conceptual information necessary for interpreting more recent findings. The search strategy combined controlled vocabulary and free-text terms related to aquaculture and edible fungi. The principal search terms included combinations of the following keywords: “*aquaculture*”, “*fish*”, “*finfish*”, “*diet*”, “*aquafeed*”, “edible fungi”, “mushroom”, “*Pleurotus*”, “*Lentinula*”, “*Ganoderma*”, “*Agaricus*”, “mycoprotein”, “fungal protein”, and “mushroom by-products”, combined using Boolean operators (AND/OR) according to the requirements of each database. Database-specific search syntax was adapted while maintaining equivalent conceptual coverage.

### 2.2. Eligibility Criteria

Studies were considered eligible if they were original peer-reviewed research articles reporting in vivo feeding trials conducted in fish species used in commercial aquaculture. Eligible studies evaluated the dietary inclusion of edible fungi or fungal-derived ingredients incorporated into aquafeeds and assessed at least one relevant outcome related to growth performance, feed utilization, physiological, immunological, antioxidant, intestinal, microbiological, or overall health responses. Studies conducted exclusively in terrestrial animals, *in vitro* experiments without in vivo validation, and investigations focused solely on fungal cultivation, mushroom production, or food technology without applications in aquaculture were excluded. Conference abstracts, editorials, theses, patents, and articles lacking sufficient methodological information for critical evaluation were also excluded. Review articles were not included in the qualitative synthesis; however, they were consulted to support the interpretation of biological mechanisms, provide conceptual background, and identify current research trends.

### 2.3. Study Selection

**Figure 1:**
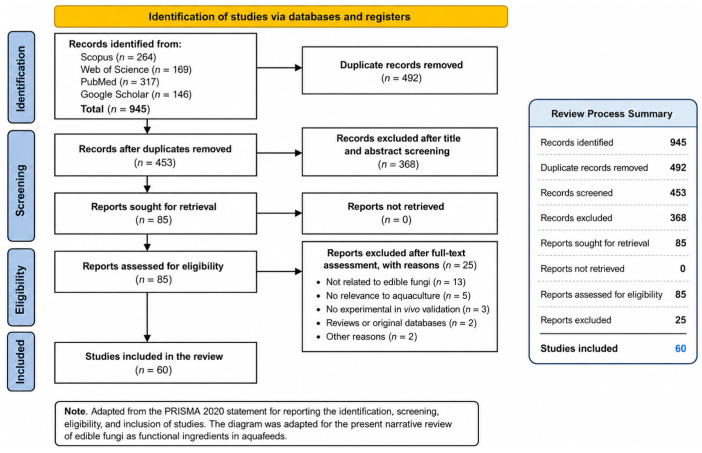
PRISMA 2020 flow diagram illustrating the study selection process.

All references retrieved from the four databases were exported to Zotero, where duplicate records were identified and removed. The study selection process was conducted in two sequential stages: an initial screening of titles and abstracts, followed by a full-text assessment of the remaining publications. Study selection followed the Preferred Reporting Items for Systematic Reviews and Meta-Analyses (PRISMA 2020) guidelines [37], and the selection process is summarized in Figure 1. A total of 945 records were initially identified through the database search. After the removal of duplicate records, 453 unique publications remained for title and abstract screening. Of these, 368 records were excluded because they did not meet the predefined eligibility criteria. The remaining 85 publications underwent full-text evaluation, and studies that fulfilled the inclusion criteria were subsequently incorporated into the qualitative synthesis.

### 2.4. Data Extraction

For each eligible study, data were systematically extracted on fish species, fungal species, type of fungal ingredient (whole mushroom, fungal biomass, extract, polysaccharide, mycelium, fermented substrate, or mushroom by-product), reported dietary inclusion level, feeding period, and experimental conditions. Information related to growth performance, feed utilization, immune responses, antioxidant parameters, intestinal morphology, gut microbiota responses, disease resistance, principal findings, and reported study limitations was also collected. Whenever available, additional information regarding extraction procedures, processing methods, fungal matrix, structural characterization of bioactive compounds, and the physicochemical properties of the ingredients was recorded to facilitate comparison among studies and support the interpretation of the reported biological responses.

### 2.5. Evidence Assessment

Owing to the substantial heterogeneity among studies with respect to fish species, fungal ingredients, ingredient processing methods, dietary inclusion levels, feeding duration, experimental conditions, and biological endpoints, a quantitative meta-analysis was not considered appropriate. Instead, the available evidence was critically synthesized through a qualitative approach based on the consistency of findings across independent studies, biological plausibility, experimental replication, species-specific digestive physiology, characterization of fungal ingredients, reported dietary inclusion levels, and commercial applicability. The overall strength of the available evidence was qualitatively classified as high, moderate, or low according to the consistency of independent findings, the number of available in vivo studies, the quality of ingredient characterization, the evaluation of reported dietary inclusion levels, the reproducibility of biological responses, their biological plausibility, and their relevance to commercial aquaculture systems. The criteria used for this qualitative assessment of the evidence are summarized in Table 4. Illustrations were created by hand to outline and highlight the key points of our review; these were then processed using the free and open-source software Inkscape (Version 1.4.2).

## 3. Demand and Challenges in the Use of Ingredients for Commercially Important Fish

Aquafeed formulation for commercially relevant species is increasingly constrained by the need to sustain high production levels without compromising host health or system sustainability [1,36]. In species such as *Oncorhynchus mykiss*, *Salmo salar*, and *Oreochromis* spp., this pressure has driven a shift from growth-oriented paradigms toward approaches that prioritize intestinal functionality, metabolic efficiency, and physiological stability under intensive farming conditions [11,38,39]. A persistent structural bottleneck is the reliance on fishmeal and fish oil, whose limited availability and price volatility impose concurrent pressures on marine ecosystems and the economic viability of aquaculture production [1,3,40].

Although the incorporation of plant-derived ingredients has reduced this dependency, high inclusion levels are frequently associated with constraints affecting both productive performance and intestinal health [41,42,43,44]. These constraints are primarily attributed to antinutritional factors such as saponins, phytates, and lectins, which can impair digestibility, nutrient absorption, and epithelial integrity [43,45]. In carnivorous fish, these effects may manifest as disruption of the intestinal barrier, subclinical inflammation, and reduced feed efficiency, ultimately affecting growth performance and resilience under farming-related stress conditions [26,43,46]. However, the available evidence remains heterogeneous, as technological strategies including fermentation, extrusion, and enzymatic treatments can partially mitigate these adverse effects, although they do not completely eliminate the limitations associated with high dietary inclusion of plant ingredients [33,47].

These nutritional constraints are further compounded by environmental externalities linked to crop production, including deforestation, intensive water use, and competition with human food systems, thereby extending sustainability considerations beyond feed formulation to the broader agroecological context [48,49]. Accordingly, the current challenge is not simply to replace conventional ingredients, but rather to develop aquafeeds capable of maintaining productive performance under commercial farming conditions while supporting host physiological stability and efficient nutrient utilization [11,26]. From an applied perspective, candidate ingredients should be evaluated according to their effects on feed conversion efficiency, digestive tolerance, and resilience to production-related stressors, rather than solely on their proximate composition [11,13,26,35].

Within this context, increasing attention has been directed toward alternative feed ingredients, including insects, algae, yeasts, and edible fungi, not only because of their nutritional composition but also because they contain bioactive compounds capable of modulating physiological responses [50,51,52,53]. Nevertheless, these alternatives should not be regarded as functionally equivalent, since their effectiveness depends on ingredient characteristics, processing methods, dietary inclusion levels, and species-specific physiological responses under farming conditions [54,55].

Based on the currently available evidence, edible fungi represent a particularly promising alternative because they combine valuable nutritional characteristics with bioactive structural polysaccharides, including β-glucans and chitin-derived compounds, that have been associated with improvements in immune competence, antioxidant capacity, and intestinal homeostasis [14,56]. Their potential value therefore extends beyond nutrient composition to their contribution to feed functionality and production efficiency under representative aquaculture conditions. Consequently, contemporary aquaculture nutrition is progressively moving toward performance-oriented formulation frameworks in which ingredient evaluation incorporates productive performance, physiological responses, animal health, and environmental sustainability as complementary criteria for the development of functional aquafeeds.

## 4. Biological Mechanisms of Fungal-Derived Compounds in Fish

The intensification of aquaculture systems has increased the incidence of infectious diseases, establishing them as a major constraint on productivity and sectoral sustainability [1,57]. In parallel, the recurrent use of antibiotics has been widely questioned because of its contribution to antimicrobial resistance and its implications for food safety and environmental sustainability [8,58], thereby promoting the development of nutritional strategies aimed at supporting host immune responses [11,18]. Within this framework, structural polysaccharides derived from edible fungi, particularly β-glucans and chitin, have been extensively investigated because of their immunomodulatory properties [14,15,59]. These compounds are recognized by pattern-recognition receptors (PRRs), including C-type lectin receptors and Toll-like receptors, initiating conserved intracellular signaling cascades that regulate cytokine production and antimicrobial mediators. As a result, they enhance phagocytic activity, respiratory burst, and innate immune enzyme activity [11,60,61].

Importantly, the biological activity of fungal β-glucans is strongly influenced by their molecular structure. β-(1→3)-glucans with β-(1→6) branches, which predominate in edible fungi, exhibit different receptor-binding properties and immunological activities compared with cereal β-(1→3)/(1→4)-glucans or bacterial glucans [30,62]. Furthermore, structural characteristics including glycosidic linkage type, degree of branching, molecular weight, particle size, and solubility influence receptor recognition, bioavailability, and downstream biological responses, contributing to the variability observed among studies [30,62]. Consequently, differences in extraction procedures, purification methods, and fungal species may substantially affect the functional properties of the resulting bioactive compounds. Evidence from yeast-derived β-glucans also supports their immunomodulatory activity in fish; however, yeast and macrofungal β-glucans should not be regarded as structurally or functionally interchangeable [63].

**Figure 2:**
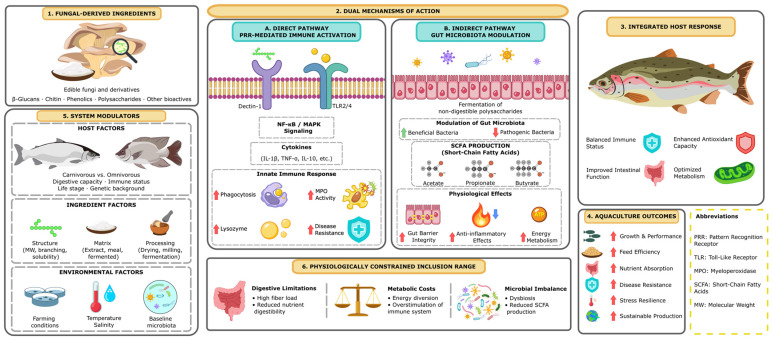
Integrated mechanism of action of fungal-derived compounds in aquaculture (PRR–microbiota–SCFA–performance axis).

Beyond acute immune activation, fungal β-glucans have also been associated with sustained modulation of innate immune responses, potentially involving longer-lasting changes in immune cell responsiveness and gene expression [16,61,64,65]. Although these mechanisms are increasingly recognized, their persistence and physiological significance in fish require further investigation. Accordingly, the biological effects of fungal-derived compounds should not be interpreted as the consequence of isolated signaling pathways but rather as the result of coordinated interactions among immune activation, intestinal physiology, microbial metabolism, and nutrient utilization within the digestive tract, collectively shaping the host response (Figure 2) [13,24,25,66].

## 5. Edible Fungi as Nutritional and Sustainable Ingredients in Aquaculture

Edible fungi have gained increasing attention in aquaculture nutrition because of their ability to provide both nutritional value and biological functionality [14,56,67]. Species such as *Pleurotus ostreatus*, *Lentinula edodes*, and *Ganoderma lucidum* exhibit moderate protein content and a compositional profile that includes essential amino acids, B-complex vitamins, trace minerals, and structurally diverse polysaccharides with biological activity [67,68,69]. These characteristics position edible fungi as promising ingredients for aquafeed formulation, particularly within nutritional strategies that aim to combine nutrient supply with functional benefits for the host [70,71,72]. However, their nutritional value is intrinsically influenced by structural polysaccharides such as β-glucans and chitin, which constitute a substantial proportion of the cell wall and may reduce nutrient digestibility, particularly in carnivorous fish species [43,73,74].

Consequently, the nutritional value of edible fungi should be interpreted as the result of a balance between their functional bioactive components and their potential effects on nutrient utilization. At elevated dietary inclusion levels, this balance may become less favorable, resulting in reduced feed utilization efficiency and, in some studies, decreased growth performance [43,68,74]. From a technological perspective, edible fungi are available in a wide variety of forms, including mushroom meals, whole biomass, cultivation by-products, fermented substrates, purified polysaccharide fractions, and enriched extracts [14,75,76,77]. Each form exhibits distinct compositional, nutritional, and functional characteristics that directly influence its biological performance under aquaculture conditions [75,76,77]. Likewise, processing methods such as drying, extraction, fermentation, and substrate selection may substantially modify the concentration, bioavailability, and biological activity of fungal bioactive compounds, contributing to the variability reported among studies [33,77,78].

**Figure 3:**
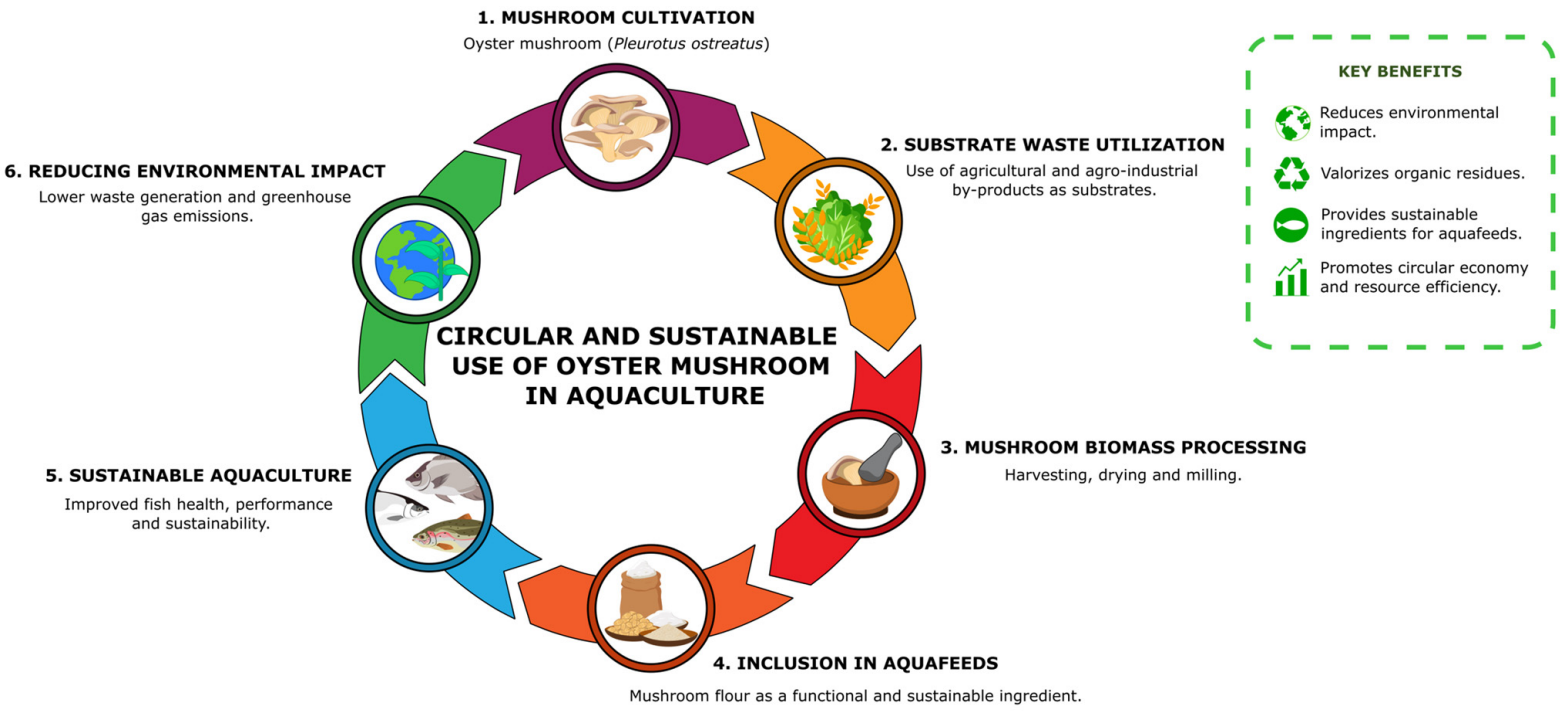
Circular bioeconomy framework of oyster mushroom (*Pleurotus ostreatus*) utilization in aquaculture.

An additional advantage of edible fungi lies in their sustainability profile, particularly their capacity to be cultivated on lignocellulosic agro-industrial residues (Figure 3) [1,75,79]. Production on substrates such as wheat straw, sugarcane bagasse, and other agricultural by-products promotes waste valorization through bioconversion processes while generating fungal biomass with potential application in aquafeeds [79]. Nevertheless, the sustainability advantages associated with fungal cultivation do not automatically translate into nutritional consistency or commercial applicability. Variability in substrate composition, cultivation conditions, fungal species, and processing methods may result in considerable differences in ingredient composition and functional properties, thereby limiting product standardization [76,80]. In addition, relatively few studies have evaluated edible fungi under commercial farming conditions, and the economic feasibility of large-scale production remains insufficiently documented [14,81,82].

Accordingly, the practical implementation of edible fungi as aquafeed ingredients depends on their ability to maintain productive performance under intensive farming conditions while simultaneously providing nutritional, physiological, and environmental benefits [1,3,4]. Based on the currently available evidence, edible fungi should be regarded as complementary functional ingredients rather than universal replacements for conventional protein sources. Their greatest potential lies in performance-oriented formulation strategies, where they may contribute to improved feed utilization, physiological resilience, and the overall sustainability of aquaculture production systems [13,34,83].

## 6. Gut Microbiota and Modulation by Functional Fungal Ingredients

The gut microbiota constitutes a central component of fish physiology, influencing digestive efficiency, intestinal stability, resilience to environmental perturbations, and host energy homeostasis [27,46,83,84,85]. The intestinal microbial community, typically dominated by members of the phyla *Proteobacteria*, *Firmicutes*, *Bacteroidetes*, and *Actinobacteria*, is highly dynamic and is shaped by host species, developmental stage, diet, and farming conditions [46,86]. Among these factors, diet remains one of the principal drivers of microbial community composition and metabolic activity [46,83]. Consequently, fungal-derived ingredients have attracted increasing attention because of their potential to influence intestinal microbial communities and their metabolic functions [14,18,77]. Nevertheless, the available evidence remains heterogeneous, and both the magnitude and direction of the reported responses vary considerably among studies. Some studies have reported increases in bacterial taxa commonly associated with beneficial intestinal functions, including *Lactobacillus* and *Bacillus*, together with improvements in intestinal morphology, digestive efficiency, and nutrient utilization [87,88,89]. However, these responses are not consistently observed and frequently depend on fungal species, ingredient type, processing method, dietary inclusion level, feeding duration, and host species. This variability highlights the context-dependent nature of microbiota modulation. Furthermore, differences in sequencing methodologies, bioinformatic pipelines, and microbiome diversity metrics may also contribute to discrepancies among published studies, thereby limiting direct comparisons [18,90,91]. Accordingly, microbial modulation should not be interpreted as a universal property of edible fungi but rather as a biological response that depends on interactions between fungal-derived ingredients, diet composition, and the pre-existing intestinal microbial ecosystem [13,19,83].

Current evidence also suggests that improvements in gut microbiota composition do not necessarily translate into proportional improvements in growth performance or health status, particularly under conditions of intestinal dysbiosis or environmental stress, where host physiological responses may override dietary effects [46,83,92]. Consequently, the application of edible fungi as functional ingredients should be interpreted within a multifactorial framework, in which microbial modulation represents one of several complementary mechanisms contributing to host performance. Future studies should prioritize standardized experimental protocols, harmonized microbiome analyses, and validation under commercial farming conditions to improve reproducibility and facilitate the practical application of fungal-derived ingredients in aquaculture.

## 7. Application of Edible Fungi in Diets for Commercial Fish

### 7.1. Rainbow Trout (Oncorhynchus mykiss)

Trophic strategy represents a central determinant of fish responses to fungal-derived ingredients because it influences digestive physiology, metabolic capacity, and interactions between diet and the intestinal microbiota [26,46,74]. Under this framework, the utilization of structural polysaccharides depends not only on their chemical composition but also on their compatibility with host digestive and metabolic characteristics, ultimately determining their biological fate within the gastrointestinal tract [26,43,46]. Carnivorous species such as *Salmo salar* and *Oncorhynchus mykiss* exhibit a limited capacity to digest and ferment complex structural polysaccharides, making them more sensitive to dietary fibre-rich ingredients [43,74,93]. This limitation has been associated with reduced carbohydrase activity and a comparatively lower microbial capacity to utilize complex carbohydrates, thereby restricting their nutritional utilization [43,46,94]. From a physiological perspective, this may also limit the production of microbial metabolites, including short-chain fatty acids, although direct evidence in fish remains limited [19,83]. Studies in *Oncorhynchus mykiss* indicate that mushroom meals derived from *Agaricus bisporus*, *Lentinula edodes*, and *Pleurotus ostreatus* can be incorporated at the dietary inclusion levels evaluated without compromising growth performance or protein digestibility [77,95,96]. The principal *in vivo* studies evaluating edible fungi and fungal-derived compounds in salmonids are summarized in Table 1.

Nevertheless, some studies have reported reductions in lipid digestibility or increased bile acid excretion at higher dietary inclusion levels, suggesting physiological constraints associated with excessive structural polysaccharide intake [43,103]. These findings indicate that digestive tolerance depends on both ingredient characteristics and host physiology rather than on dietary fibre concentration alone [43,74,103]. In contrast, omnivorous species such as *Oreochromis niloticus* appear to exhibit greater metabolic flexibility and a higher capacity to utilize complex carbohydrates, according to the currently available evidence [26,46,104]. In these species, low to moderate dietary inclusion of edible fungi has frequently been associated with improvements in growth performance, feed utilization, antioxidant status, and innate immune responses [54,78,104]. These responses are likely related to a greater capacity of the intestinal microbiota to metabolize complex polysaccharides and generate metabolites that contribute to intestinal homeostasis, although the underlying mechanisms remain incompletely understood [16,24,105].

Overall, the available evidence indicates that responses to fungal-derived ingredients are highly species-dependent and are influenced by digestive physiology, gut microbiota composition, ingredient characteristics, processing methods, and dietary inclusion level [19,26,46,55]. Rather than assuming a universal dose–response relationship, current evidence supports the existence of species-specific responses that should be interpreted within the experimental conditions evaluated in each study. Consequently, omnivorous species generally appear to tolerate a broader range of dietary fungal ingredients, whereas carnivorous fish may require lower dietary inclusion levels and more targeted formulations to maximize potential functional benefits while minimizing adverse effects on nutrient utilization and digestive efficiency [26,43,46]. These differences highlight the importance of developing species-specific formulation strategies instead of directly extrapolating nutritional responses across fish with contrasting trophic habits [11,26,43,46].

### 7.2. Atlantic Salmon (Salmo salar)

The use of functional ingredients in diets for Atlantic salmon (*Salmo salar*) has gained increasing attention as a strategy to support intestinal health, maintain productive performance, and reduce dependence on conventional feed inputs in intensive aquaculture systems [1,3,106]. Within this context, fungal-derived bioactive compounds have attracted interest because of their potential to modulate physiological processes associated with innate immunity, oxidative status, and intestinal function, extending beyond their nutritional contribution [8,11,106]. However, direct evidence evaluating edible fungi in diets for *Salmo salar* remains scarce, limiting the development of species-specific nutritional recommendations [14,77]. Consequently, much of the current understanding is derived from studies conducted in other salmonid species or from investigations using functionally related fungal-derived ingredients. Although these studies provide useful physiological insights, their findings should not be directly extrapolated to Atlantic salmon without species-specific validation [11,19,83].

Studies in related salmonids illustrate this potential. In *Oncorhynchus masou*, dietary supplementation with *Pleurotus ostreatus*-derived products has been associated with improvements in digestibility and intestinal health under the experimental conditions evaluated, whereas higher dietary inclusion levels have been associated with reduced productive performance [14,101]. Likewise, in *Oncorhynchus kisutch*, ergothioneine-rich fungal extracts have demonstrated antioxidant activity that may contribute to improved product quality and oxidative stability [102]. Collectively, these findings support the potential of fungal-derived ingredients in salmonids while also indicating that their biological responses are influenced by ingredient type, processing method, dietary inclusion level, and host species [101,102,107].

For Atlantic salmon, the principal limitation is not necessarily the absence of beneficial biological effects but the limited availability of studies conducted under commercial farming conditions using edible fungal ingredients [77,82,106]. This limitation is particularly relevant considering the digestive physiology of *Salmo salar*, which exhibits a relatively limited capacity to utilize structural polysaccharides, potentially restricting the efficient use of whole fungal biomass [14,43]. Accordingly, processed fungal ingredients, purified extracts, or specific bioactive fractions may represent more suitable alternatives than unprocessed fungal meals, although this hypothesis requires experimental confirmation in Atlantic salmon [14,55,62]. Based on the currently available evidence, edible fungi should be regarded as promising complementary functional ingredients rather than direct substitutes for conventional protein sources in diets for *Salmo salar*. Future research should prioritize integrated evaluations of productive performance, intestinal health, immune responses, oxidative status, and economic feasibility under representative commercial farming conditions. Such studies will be essential to determine the practical applicability of edible fungi in Atlantic salmon production systems and to establish evidence-based dietary inclusion strategies [54,80].

### 7.3. Application of Edible Fungi in Diets for Tilapia (Oreochromis spp.)

Tilapia (*Oreochromis* spp.) represents one of the most widely studied models for evaluating functional ingredients in aquaculture because of its rapid growth, broad physiological plasticity, and adaptability to intensive farming systems [2]. These characteristics, together with its global importance for aquaculture production, have promoted the development of nutritional strategies aimed not only at sustaining growth but also at supporting intestinal health and physiological resilience under farming-related stress [2,11,108]. Edible fungi have been extensively investigated in this species because of their content of bioactive polysaccharides and other metabolites with immunomodulatory and antioxidant potential [15,78,109]. Compared with salmonids, the evidence available for tilapia is more extensive, allowing a more comprehensive evaluation of productive, immunological, and physiological responses [54,78].

As summarized in Table 2, many studies have reported improvements in growth performance and feed utilization following dietary supplementation with edible fungi, although the magnitude of these responses varies according to fungal species, ingredient type, processing method, dietary inclusion level, and experimental conditions [54,78,110]. At higher dietary inclusion levels, some studies have reported reductions in productive performance, indicating that biological responses are not necessarily proportional to increasing dietary inclusion [78,110]. The mechanisms underlying these responses are likely multifactorial. Current evidence suggests that the intestinal microbiota may contribute to the utilization of fungal structural polysaccharides and the production of biologically active metabolites, although direct mechanistic evidence remains limited [19,26,74]. Likewise, the greater digestive plasticity of omnivorous fish may facilitate the utilization of fungal-derived ingredients compared with carnivorous species [16,24].

At the physiological level, fungal-derived ingredients have frequently been associated with improvements in antioxidant status, innate immune responses, and intestinal function [14,54,78]. Dietary supplementation has been associated with increased activity of antioxidant enzymes, including superoxide dismutase and catalase, together with improved tolerance to oxidative and environmental stress [54,78,115]. Likewise, increases in lysozyme activity, peroxidase activity, and phagocytic capacity have been reported in several studies, suggesting stimulation of innate immune responses beyond their nutritional contribution [88,113,115]. These effects are consistent with the proposed recognition of fungal polysaccharides by pattern recognition receptors, although the relative contribution of this mechanism may differ among fungal ingredients and host species [11,83].

At the intestinal level, some studies have reported increases in bacterial taxa commonly associated with intestinal health following dietary supplementation with fungal-derived ingredients, together with improvements in intestinal morphology and epithelial integrity [15]. However, these responses are not universal and appear to depend on ingredient characteristics, dietary formulation, host species, and experimental conditions, consistent with the multifactorial regulation of the intestinal microbiota.

Despite these overall trends, responses are not entirely uniform across studies or fungal-derived compounds. Improvements in innate immune responses are not always accompanied by comparable changes in humoral immune parameters, indicating that ingredient type, processing, dietary inclusion level, and formulation influence both the magnitude and nature of the physiological response [15,112,116]. Overall, current evidence indicates that the biological effects of edible fungi in *Oreochromis* spp. arise from coordinated interactions among digestive physiology, intestinal microbiota, metabolism, and immune function rather than from dietary inclusion level alone [19,28,83]. Accordingly, edible fungi should be regarded as complementary functional ingredients whose effectiveness depends on the nutritional and physiological context in which they are applied.

Among the fish species evaluated to date, tilapia currently represents one of the strongest evidence bases supporting the use of edible fungi as functional aquafeed ingredients, although additional studies under commercial farming conditions remain necessary to confirm their long-term applicability [14,54,78,104].

## 8. Methodological Gaps: Dosage, Matrices, and Processing

The current evidence on the use of β-glucans and edible fungal-derived ingredients in aquaculture is characterized by substantial methodological heterogeneity, which limits cross-study comparability and constrains the translation of experimental findings to commercial production systems [28,107]. This variability extends beyond individual experimental differences and reflects the absence of standardized approaches for ingredient characterization, experimental design, and biological evaluation [11,28]. A major source of inconsistency lies in the wide range of dietary inclusion levels investigated. Studies encompass purified β-glucans administered at very low concentrations as well as whole fungal biomass incorporated at substantially higher dietary levels, resulting in marked differences in the quantity and physicochemical characteristics of the bioactive compounds being evaluated [14,54]. Consequently, dietary inclusion level alone does not provide a reliable basis for comparing biological responses among studies. Instead, interpretation requires detailed information on ingredient composition, bioactive compound concentration, and physicochemical properties that determine functional availability [11,14,24].

Methodological variability is further increased by the diversity of fungal ingredient matrices evaluated, including mushroom meals, whole biomass, cultivation by-products, fermented substrates, cell-wall fractions, purified polysaccharides, and enriched extracts [14,94]. These ingredients differ not only in their chemical composition but also in their structural characteristics, degree of processing, and biological availability, making direct comparisons inherently difficult. In addition, the limited characterization of properties such as molecular weight, branching pattern, solubility, and particle size further restricts the interpretation and reproducibility of published findings [30,33,81]. Processing represents another major source of variability. Technological interventions such as drying, extrusion, fermentation, enzymatic hydrolysis, and extraction modify the physicochemical properties of fungal-derived compounds and may substantially influence their biological activity [30,33]. However, processing conditions are frequently insufficiently described, limiting experimental reproducibility and preventing rigorous comparisons among studies [28,33,107].

Additional methodological differences-including feeding duration, sample size, fish developmental stage, diet composition, water quality, temperature, stocking density, and production system—are also known to influence physiological responses but are not consistently incorporated into experimental designs [46]. Likewise, intestinal microbiota composition varies according to host species, farming environment, and management practices, introducing additional biological variability that may influence the response to fungal-derived ingredients [8,107,117]. Consequently, it is often difficult to distinguish whether observed biological responses are primarily attributable to ingredient characteristics, processing methods, dietary formulation, or production conditions. Collectively, these methodological limitations largely explain the variability reported for edible fungal ingredients across fish species and experimental systems [14,28].

Future research should prioritize standardized ingredient characterization, transparent reporting of processing methods, harmonized experimental protocols, and comprehensive evaluation of productive, immunological, intestinal, and microbiological responses. Such approaches will improve reproducibility, facilitate meaningful comparisons among studies, and strengthen the evidence base required for the practical application of edible fungi in aquaculture nutrition.

Collectively, the evidence summarized in Table 3 highlights that the biological responses to edible fungi depend on the interaction among ingredient characteristics, processing methods, host physiology, and experimental conditions. These findings underscore the need for standardized ingredient characterization, harmonized experimental protocols, and validation under commercial farming conditions before evidence-based dietary recommendations can be established.

## 9. Discussion

The available evidence indicates that edible fungi represent promising functional ingredients in aquaculture nutrition; however, their biological effects do not follow a simple proportional dose–response relationship but instead arise from the interaction of multiple physiological, nutritional, and environmental factors (Table 4) [11,77,107]. Consequently, the variability reported among studies should not be interpreted exclusively as methodological inconsistency but also as a reflection of the biological complexity underlying host responses to fungal-derived ingredients [26,107]. The evidence synthesized in this review supports a conceptual model in which the biological activity of fungal-derived ingredients is likely mediated through interacting mechanisms involving both direct immune stimulation and digestive-metabolic processes associated with their utilization within the gastrointestinal tract [61,62,83]. β-Glucans are recognized by pattern recognition receptors, promoting innate immune responses that have been reported even at relatively low dietary inclusion levels [16,61]. Conversely, when edible fungi are incorporated as whole biomass or minimally processed ingredients, their biological effects are also influenced by digestibility, ingredient composition, and interactions with the intestinal environment [14,78]. The relative contribution of these mechanisms appears to vary according to fungal species, ingredient processing, host physiology, and dietary inclusion level.

This conceptual framework helps explain why biological responses are not consistently proportional to increasing dietary inclusion. Rather, beneficial effects appear to depend on maintaining an appropriate balance between physiological stimulation and digestive utilization under the experimental conditions evaluated. When dietary inclusion exceeds the digestive capacity of the host, reductions in feed utilization efficiency and productive performance have been reported in some studies [43,110]. This tendency appears to be more evident in carnivorous fish, whereas omnivorous species generally exhibit greater digestive flexibility toward fungal-derived ingredients, although considerable variability remains among studies [41,74,104].

In addition, host responses are influenced by the initial physiological condition of the fish, including intestinal microbiota composition, nutritional status, and environmental conditions, highlighting the context-dependent nature of fungal-derived ingredients [19,46,119]. Processing also represents a major determinant of biological activity because it modifies physicochemical characteristics that influence digestibility, bioavailability, and interactions with the host [30,33]. Consequently, mushroom meals, fermented substrates, cultivation by-products, purified polysaccharides, and enriched extracts should not be regarded as functionally equivalent ingredients. From an applied perspective, the evidence currently available suggests that edible fungi are best regarded as complementary functional ingredients whose effectiveness depends on the interaction among ingredient characteristics, processing methods, host digestive physiology, intestinal microbiota, and farming conditions rather than on dietary inclusion level alone.

## 10. Conclusions

Edible fungi represent promising complementary functional ingredients in aquaculture nutrition, with the potential to support intestinal function, innate immune responses, and physiological homeostasis beyond their nutritional contribution. The evidence synthesized in this review indicates that their biological effects arise from the interaction of multiple factors, including ingredient composition, structural characteristics, processing methods, host digestive physiology, intestinal microbiota, and farming conditions. Consequently, the available evidence does not support a universal dose–response relationship or generalized dietary inclusion recommendations across fish species. Instead, biological responses should be interpreted within the specific experimental conditions evaluated, considering both the characteristics of the fungal ingredient and those of the host species.

The variability observed among studies reflects not only methodological heterogeneity but also the biological complexity underlying host responses to fungal-derived ingredients. Differences in ingredient characterization, processing methods, experimental design, and biological endpoints continue to limit comparisons among studies and restrict the development of evidence-based strategies for their application in commercial aquaculture.

Overall, edible fungi should be regarded as complementary functional ingredients rather than direct replacements for conventional protein sources. Their greatest potential lies in their strategic incorporation into functional feeding programs aimed at improving physiological resilience and promoting sustainable aquaculture production under specific farming conditions. Future research should prioritize standardized ingredient characterization, harmonized experimental protocols, comprehensive evaluation of productive and physiological responses, and validation under commercial farming conditions. These efforts will strengthen the available evidence, improve reproducibility, and facilitate the practical implementation of edible fungi in sustainable aquaculture nutrition.

## Figures and Tables

**Table 1:** *In vivo* Studies on edible fungi and derived compounds in salmonids (Salmonidae).

Fish Species	Fungal Species	Fungal Ingredient Type	Extraction/Preparation	Dietary Inclusion	Feeding Period	Principal Biological Effects	Main Limitations	Ref.
*Oncorhynchus mykiss*	*Grifola gargal*	Whole fruiting-body meal	Fruiting bodies shredded, lyophilized and ground	25 and 100 g/kg diet	56 d	25 g/kg improved growth, feed efficiency, protein efficiency ratio, nutrient retention, liver glycogen and body lipid content, while reducing VSI.	Growth and feed utilization declined at 100 g/kg. Higher dietary inclusion levels were associated with reduced growth performance.	[95]
*Oncorhynchus mykiss*	*Pleurotus ostreatus* + *Lentinula edodes*	Fermented wheat–mycelium substrate	Wheat fermented by fungal mycelia (solid-state fermentation), dried and ground	100 g/kg diet	56 d	Improved growth, feed efficiency, nutrient retention, hepatic glycogen and phosphorus utilization; *P. ostreatus* produced the greatest improvement.	Fermented substrate rather than purified fungal biomass; only one inclusion level evaluated.	[96]
*Oncorhynchus mykiss*	*Pleurotus ostreatus*	Fruiting-body extract	40% methanolic extract	0.1 and 0.5 g/kg diet	30 d	Increased growth, respiratory burst, phagocytic activity, lysozyme and myeloperoxidase (MPO) activity; improved innate immunity.	No significant improvement in survival after *Aeromonas hydrophila* challenge compared with the control.	[20]
*Oncorhynchus mykiss*	*Pleurotus ostreatus*	Mushroom extract	Hot-water extract (60–65 °C, 24 h), filtered and lyophilized	10 and 20 g/kg diet	42 d	Enhanced innate immune responses, including phagocytosis, lysozyme and MPO activity; significantly improved survival following *Lactococcus garvieae* challenge.	Growth performance was not the primary endpoint.	[97]
*Oncorhynchus mykiss*	*Ganoderma applanatum*	Mushroom extract	Hot-water extract (95–100 °C), concentrated and freeze-dried	250, 500 and 1000 mg/kg diet	45 d	Increased serum proteins, leukocyte counts, lysozyme activity and non-specific immune responses, particularly at 1000 mg/kg.	No significant growth improvement was observed.	[98]
*Oncorhynchus mykiss*	*Pleurotus ostreatus*	Mushroom extract	Hot-water extract (60–65 °C for 24 h), filtered and lyophilized	10 and 20 g/kg diet (1 and 2%)	42 d	Increased total leukocyte, neutrophil and monocyte counts, phagocytic activity, lysozyme, MPO, serum protein and globulin without adverse intestinal histological changes.	Growth performance and feed conversion ratio were not significantly affected; no pathogen challenge was performed.	[99]
*Oncorhynchus mykiss*	*Lentinula edodes*	Mushroom extract	Hot-water extract (100 g dried mushroom/200 mL water; 60–65 °C for 24 h), filtered and lyophilized	10 and 20 g/kg diet (1 and 2%)	42 d	Increased total leukocyte, neutrophil and monocyte counts, phagocytic activity, lysozyme, MPO and serum IgM (2% diet), resulting in significantly higher survival after *Lactococcus garvieae* challenge (RPS = 55%).	Growth performance was not evaluated because the study focused on immunological responses and disease resistance under commercial farming conditions.	[100]
*Oncorhynchus mykiss*	*Agaricus bisporus*, *Lentinula edodes* and *Pleurotus ostreatus*	Mushroom by-product meals	Dried stems, broken and non-commercial mushrooms	300 g/kg diet	42 d	Growth performance was maintained; apparent digestibility coefficients for dietary protein exceeded 90%, and mushroom protein digestibility exceeded 77%.	Digestibility trial only; diets were not formulated to evaluate long-term production performance.	[77]
*Oncorhynchus masou masou*	*Pleurotus ostreatus*	Mushroom by-product meal	Dried mushroom by-product incorporated directly into feed	35–140 g/kg diet	Not clearly reported	Growth performance was maintained at 35–70 g/kg, whereas inclusion levels ≥105 g/kg reduced feed intake and body weight gain.	Immune responses were not evaluated; feeding period was not clearly reported.	[101]
*Oncorhynchus kisutch*	*Pleurotus cornucopiae*	Ergothioneine-rich mushroom extract	Repeated hot-water extraction followed by vacuum concentration	Equivalent to approximately 550 µg ergothioneine kg^−1^ body weight day^−1^	2 months	Reduced lipid oxidation and improved astaxanthin retention during refrigerated storage, thereby enhancing fillet quality.	The study focused on post-harvest flesh quality rather than growth or immune responses.	[102]

Abbreviations: FCR, feed conversion ratio; VSI, viscerosomatic index; MPO, myeloperoxidase; Ig, immunoglobulin. “Not reported” indicates that the parameter was not specified in the original study. The expression “no growth improvement” denotes the absence of statistically significant differences in growth-related parameters compared to the control group.

**Table 2:** *In vivo* Studies on edible fungi and derived compounds in tilapia (*Oreochromis* spp.).

Fish Species	Fungal Species	Fungal Ingredient Type	Preparation/Extraction	Dietary Inclusion	Feeding Period	Principal Biological Effects	Main Limitations	Ref.
*Oreochromis niloticus*	*Pleurotus ostreatus*	Mushroom cultivation by-product meal	By-product of *P. ostreatus* cultivation on rice straw, dried and incorporated directly into diets	50, 100, 150 and 200 g/kg diet	112 d (16 weeks)	Growth performance, feed utilization and protein utilization were maintained up to 200 g/kg; dietary inclusion also improved economic efficiency.	Economic performance was evaluated, but immune and antioxidant responses were not investigated.	[111]
*Oreochromis niloticus*	*Pleurotus djamor*	Whole mushroom meal	Dried fruiting bodies ground into meal	Replacement of soybean meal at 25, 50, 75 and 100%	45 d	Inclusion levels above 25% reduced weight gain, specific growth rate, daily feed intake and lipase activity, while increasing whole-body crude fibre.	High replacement levels impaired growth performance; immune responses were not evaluated.	[110]
*Oreochromis niloticus*	*Ganoderma lucidum*	Mushroom powder	Dried reishi mushroom powder incorporated directly into diets	5, 10 and 20 g/kg diet	90 d	Improved growth, feed conversion ratio, growth-related gene expression, digestive enzyme gene expression and lipid metabolism; most favorable responses observed at ~12.5 g/kg diet.	Only juvenile Nile tilapia were evaluated; immune responses were not investigated.	[78]
*Oreochromis niloticus*	*Agaricus bisporus*	Mushroom powder	Whole white button mushroom powder incorporated into diets	5, 10, 20 and 40 g/kg diet (0.5–4%)	60 d	Improved growth, feed efficiency, lysozyme activity, antioxidant enzymes (SOD, CAT and GPx), immune-related gene expression and resistance to heat stress; most favorable responses observed at 2.15–2.75%.	Protective effects were evaluated only under experimental heat stress.	[54]
*Oreochromis niloticus*	*Agaricus bisporus*	Mushroom extract	Aqueous mushroom extract incorporated into diets	60 g/kg diet (6%)	42 d (6 weeks)	Increased lysozyme activity, alternative complement (ACH50), erythrocyte and leukocyte counts, haemoglobin and haematocrit values.	No significant improvement in immunoglobulin (Ig) levels was observed.	[112]
*Oreochromis niloticus*	*Pleurotus djamor var. roseus*	Mushroom meal	Dried mushroom meal incorporated into diets	150, 200 and 250 g/kg diet (15–25%)	60 d	Dietary inclusion at 150–200 g/kg improved weight gain, specific growth rate and hematological parameters, particularly leukocyte and lymphocyte counts.	Higher inclusion (250 g/kg) did not further improve growth, Most favorable responses observed at intermediate inclusion levels.	[113]
*Oreochromis spp.*	*Pleurotus pulmonarius*	Mushroom extract	Not clearly described in the original source	Not clearly reported	Not clearly reported	Increased antioxidant enzyme activities, particularly superoxide dismutase (SOD) and catalase (CAT).	Experimental conditions, inclusion level and extraction procedure were insufficiently described.	[107]
*Oreochromis niloticus*	*Pleurotus* spp. (mushroom stalk waste)	Hot-water extract	Hot-water extract (2 or 5 h) obtained from mushroom stalk waste; 5 h extract showed higher β-glucan and antioxidant activity	5 and 10 g/kg diet	Not clearly reported	Dietary supplementation with 5 g/kg improved growth performance and antioxidant enzyme activities (SOD and CAT); the 5 h extract was more effective than the 2 h extract.	Study focused on antioxidant status; immune parameters were not evaluated.	[114]
*Oreochromis niloticus*	Mushroom-derived residual polysaccharides	Fungal polysaccharide preparation	Residual polysaccharides isolated from mushroom processing by-products	5–100 g/kg diet	Not reported	Improved growth performance, antioxidant capacity and innate immune responses in experimental studies summarized in the literature.	Evidence derived from multiple studies; extraction methods and experimental protocols varied considerably.	[115]
*Oreochromis niloticus*	*Cordyceps militaris*	Spent mushroom substrate (SMS)	Spent mushroom substrate incorporated alone or combined with *Lactobacillus plantarum*	10 g/kg SMS (alone or combined with 10^8^ CFU g^−1^ *L. plantarum*)	56 d (8 weeks)	Improved growth performance, feed conversion ratio, skin mucus immunity, serum lysozyme, complement activity, phagocytosis, respiratory burst and peroxidase activity; combined supplementation produced the strongest response.	The synergistic effect of SMS with probiotics makes it difficult to distinguish the contribution of the fungal substrate alone.	[88]

Abbreviations: SOD, superoxide dismutase; CAT, catalase; Ig, immunoglobulin; FM, fish meal. “Not reported” indicates that the parameter was not specified in the original study. The expression “no growth improvement” denotes the absence of statistically significant differences in growth-related parameters compared to the control group.

**Table 3:** Reported functional inclusion ranges and evidence levels of edible fungi and fungal-derived ingredients evaluated in aquaculture species.

Group/Species	Functional Ingredient	Reported Effective Inclusion Range (g/kg diet)	Highest Tested Inclusion Associated with Adverse Responses	Principal Reported Benefits	Reported Limitations	Overall Evidence
**Salmonids** (*Oncorhynchus mykiss*, *Salmo salar*)	Purified fungal extracts (β-glucans, polysaccharides and aqueous/methanolic extracts)	**0.1–20**	>20 (limited evidence)	Enhanced innate immunity, antioxidant capacity and disease resistance	Responses depend on extract type, dosage and feeding duration; evidence for long-term feeding remains limited	**High**
	Mushroom meals and fermented substrates	**25–150**	≥200	Improved growth, feed utilization, nutrient retention and body composition at moderate inclusion levels	Reduced digestibility and excessive dietary fibre at high inclusion levels	**Moderate–high**
**Related salmonids** (*Oncorhynchus masou*, *Oncorhynchus kisutch*)	Mushroom meal	**35–70**	≥105	Maintained growth performance at moderate dietary inclusion levels	High dietary inclusion reduced feed intake and growth performance	**Moderate**
**Tilapia** (*Oreochromis niloticus*)	Mushroom powders and meals	**50–250**	≥500	Improved growth, feed efficiency, antioxidant status and immune response	Excessive inclusion may reduce nutrient digestibility and alter carcass composition	**High**
	Mushroom extracts	**5–15**	>20	Enhanced antioxidant capacity, innate immunity and stress tolerance	Additional benefits plateau at higher inclusion levels	**High**
	Mushroom by-products (stems, cultivation residues, spent substrates)	100–300 (or partial fishmeal/soybean meal replacement)	>300	Sustainable protein source with acceptable growth performance and immune support	Variable nutritional composition depending on mushroom species and processing method	**Moderate**
**Catfish** (*Clarias gariepinus*)	Mushroom stem meal	**25–75**	≥100	Improved intestinal morphology and villus development	Limited evidence for improvements in growth performance	**Low**

“Not reported” indicates that the parameter was not specified in the original study. Consistency of outcomes was qualitatively classified based on the reproducibility and agreement of results across measured variables. Evidence level was determined considering experimental design, sample size, duration, and relevance to production conditions.

**Table 4:** Qualitative evaluation of functional inclusion ranges and evidence levels of fungal-derived ingredients in aquaculture species.

Study	Species	Fungal Ingredient	Experimental Design	Feeding Period (Days)	Main Endpoints Evaluated	Consistency of Findings	Level of Evidence
Pascual et al., 2017 [95]	*Oncorhynchus mykiss*	Whole fruiting-body meal of *Grifola gargal*	3 dietary treatments × 3 replicate tanks × 50 fish per tank	56	Growth performance, feed intake and utilization, nutrient retention, whole-body composition and somatic indices	Dose-dependent: 25 g/kg consistently improved growth and nutrient utilization, whereas 100 g/kg impaired performance	Moderate
Pascual et al., 2018 [96]	*Oncorhynchus mykiss*	Wheat grains fermented by *Pleurotus ostreatus* or *Lentinula edodes mycelia*	3 dietary treatments × 3 replicate tanks × 50 fish per tank	56	Growth performance, feed efficiency, nutrient retention, whole-body composition, liver glycogen and somatic indices	Moderate-to-high: both fermented substrates improved performance, although effects differed between fungal species	Moderate
Saromines et al., 2025 [77]	*Oncorhynchus mykiss*	By-product meals from *Agaricus bisporus, Lentinula edodes* and *Pleurotus ostreatus*	*In vitro* protein-hydrolysis assays plus 4 dietary treatments × 3 replicate tanks; 30% test-ingredient inclusion	42	*In vitro* protein hydrolysis, *in vivo* apparent digestibility coefficients, growth, feed utilization, somatic indices, blood biochemistry, body composition, digestive enzymes and liver histology	Moderate: protein digestibility was generally acceptable and growth was unaffected, but species-specific differences occurred in nutrient digestibility and hepatic responses	Moderate–high
Uluköy et al., 2016 [97]	*Oncorhynchus mykiss*	Hot-water extract of *Pleurotus ostreatus*	3 dietary treatments × 2 replicate ponds × 60 fish per replicate; subsequent *Lactococcus garvieae* challenge	42	Hematological profile, phagocytic activity, lysozyme, myeloperoxidase, total immunoglobulin and post-challenge mortality	High for innate immune responses: most hematological and immune variables improved and mortality decreased, although total immunoglobulin was unaffected	Moderate–high
Bilen et al., 2016 [20]	*Oncorhynchus mykiss*	40% methanolic extract of *Pleurotus ostreatus*	5 dietary groups × 3 replicate tanks × 30 fish per tank; oyster mushroom and nettle extracts evaluated separately; subsequent *Aeromonas hydrophila* challenge	30	Growth performance, feed utilization, respiratory burst, phagocytosis, lysozyme, myeloperoxidase and post-challenge survival	Moderate: growth and innate immune variables improved, but oyster mushroom extract did not significantly increase survival relative to the control	Moderate
Yilmaz et al., 2023 [78]	*Oreochromis niloticus*	Reishi mushroom powder (*Ganoderma lucidum*)	4 dietary treatments with 3 replicate units per treatment	90	Growth, feed conversion, survival and expression of genes related to growth, appetite, digestion and lipid metabolism	High: responses consistently favoured the intermediate dose, with an estimated optimum of approximately 12.5 g/kg	Moderate–high
Dawood et al., 2020 [54]	*Oreochromis niloticus*	White button mushroom powder (*Agaricus bisporus*)	5 dietary treatments × 3 replicate aquaria × 15 fish per aquarium; total *n* = 225; subsequent heat-stress exposure	60	Growth, feed utilization, hematology, serum immunity, antioxidant status, immune- and stress-related gene expression and heat-stress resistance	High: growth, antioxidant and immune responses generally converged on an intermediate optimum, and supplemented fish showed improved stress tolerance	High
Van Doan et al., 2017 [88]	*Oreochromis niloticus*	*Cordyceps militaris* spent mushroom substrate, alone or combined with *Lactobacillus plantarum*	4 dietary treatments: control, SMS, probiotic and SMS + probiotic; replicated feeding trial	60	Growth, feed conversion, survival, skin-mucus immunity, serum lysozyme, complement, phagocytosis, respiratory burst and peroxidase activity	Moderate-to-high: the combined treatment produced the strongest and most consistent responses, but the fungal contribution cannot be separated completely from the probiotic effect	Moderate–high
Song et al., 2021 [89]	*Apostichopus japonicus*	*Pleurotus ostreatus* polysaccharides (POPS)	4 dietary treatments × 3 replicate tanks × 25 animals per tank; total n = 300	60	Growth, visceral-wall ratio, antioxidant and innate immune enzymes, gut microbial diversity and community composition	Moderate: immune and microbiota responses were dose-dependent, but growth was not significantly affected	Moderate
Hao et al., 2024 [118]	*Plectropomus leopardus*	Commercial β-glucan; biological source not specified	5 dietary treatments × 3 replicate tanks × 20 fish per tank; total n = 300	56	Growth, digestive enzymes, intestinal morphology, hepatic and serum antioxidant status, innate immunity and gene expression	High within the study: 0.10% produced the most consistent benefits, whereas 0.20% weakened growth and several physiological responses	Low for edible-fungi-specific evidence
Oh et al., 2019 [101]	*Oncorhynchus masou masou*	Dried *Pleurotus ostreatus* meal	5 dietary treatments containing 0, 3.5, 7.0, 10.5 or 14.0% mushroom meal; replication insufficiently described	28	Body weight, feed intake, feed conversion and hepatosomatic index	Variable: 3.5–7.0% produced responses comparable with the control, whereas ≥10.5% reduced feed intake and body weight	Low–moderate

Recommended ranges were derived from a comparative analysis of available studies and should be interpreted as functional guidance rather than fixed formulation values. Evidence levels were qualitatively assigned based on study design, reproducibility of results, and relevance to production conditions.

## Data Availability

No new data were created or analyzed in this study. Data sharing is not applicable to this article.

## Abbreviations

The following abbreviations are used in this manuscript:

CAT	Catalase
FCR	Feed conversion ratio
FM	Fish meal
Ig	Immunoglobulin
MPO	Myeloperoxidase
PRR	Pattern recognition receptor
SCFA	Short-chain fatty acid
